# Ethnobotanical survey of herbs used in the management of diabetes mellitus in Southern Katanga Area/DR Congo

**DOI:** 10.11604/pamj.2018.30.218.11718

**Published:** 2018-07-18

**Authors:** Bakari Amuri, Mwamba Maseho, Lumbu Simbi, Pierre Duez, Kahumba Byanga

**Affiliations:** 1 Laboratoire de Pharmacognosie, Université de Lubumbashi (UNILU), 27 Avenue Kato, Lubumbashi, Democratic Republic of Congo; 2 Unit of Therapeutic Chemistry and Pharmacognosy, Mons (UMONS), Bât 6, Chemin du Champ de Mars 25, 7000 Mons, Belgium; 3 Laboratoire de Chimie Organique, Département de Chimie, Faculté des Sciences, Université de Lubumbashi (UNILU), Université de Democratic Republic of Congo

**Keywords:** Diabetes, medicinal plants, Ethnopharmacology, Katanga

## Abstract

**Introduction:**

Diabetes is becoming a public health burden for sub-Saharan countries due to its prevalence which is growing rapidly. Traditional medicine is more and more used to treat diabetes in RD Congo as well as in other African countries. This study was undertaken in order to list plants used in the management of diabetes by traditional healers in four agglomerations of southern area of Katanga in the Democratic Republic of Congo.

**Methods:**

Forty-nine traditional healers were randomly met and interviewed about diabetes treatment in traditional medicine. The survey concerned the plant identification, their part used, method of preparation and the route of administration. The inquest concerned also traditional medicine users.

**Results:**

Ninety-five plants from 47 families were indicated as antidiabetic. Fabaceae (24.2%), Euphorbiaceae (7.4%), Apocynaceae and Strychnaceae (4.2 each) are the more representative families. This inventory showed that the root is the most used part of the cited plants, the decoction with water as the main preparation method and the oral administration as the principal way to give antidiabetic traditional formulations.

**Conclusion:**

In Lubumbashi region, many plant species are used to treat diabetes either through traditional praticians or by anyone from well-known ancestral knowledge.

## Introduction

Diabetes mellitus is a metabolic syndrome characterized by chronic high-blood glucose concentrations resulting from defects in insulin secretion, insulin action or both and having consequences on lipids and proteins metabolism [1,2]. According to the International Federation of Diabetes (IDF) there were 415 million people in the world with diabetes in 2015 and this is projected to increase to 642 million by 2040 [3]. In the Democratic Republic of Congo the prevalence of diabetes mellitus is rapidly growing up [4,5]. From 2003 to 2013 the number of diabetic patients has increased alarmingly from 552 thousands to 1.6 million; and the proportion of people with undiagnosed diabetes may reach 75% due to resource-limited health care [6,7]. On one side the limited access to conventional drugs and health care system, the faith on ancestral culture healing practices on the other hand, bring more people to traditional medicine where herbal drugs are widely used. Traditional medicine is still the mainstay of millions Congolese as well as other Africans [8]. In light of that, we decided to collect information about the plants used traditionally in the treatment of diabetes mellitus in southern Katanga area, DRC.

## Methods

This ethnobotanical survey was realized by interviews conducted with the help of a guide-questionnaire in the town of Lubumbashi, and in the cities of Kasumbalesa, Kipushi and Likasi, in the southern area of Katanga province, in the Democratic Republic of Congo from September 2005 to July 2007, according to principles stated by the Declaration of Helsinki on personal data [9]. To be sure of the information veracity, each traditional healer was met at least three times to answer the same questions at different moments. From the collected sample, the plants pointed out as providing antidiabetic properties had been identified by their scientific name at the herbarium of Kipopo (30km far from Lubumbashi town to the North), by Professor Jean Lejoly of the Free University of Brussels.

## Results

About fifty traditional healers were visited and interviewed on their knowledge and on diabetes treatment after their assent. The data obtained from different traditional healers on their knowledge and on vegetable species used in the management of diabetes are given in Table 1 and Annex 1. Table 1 gives information about traditional practitioners (tribe, age, sex and how he or she became healer). Annex 1 gives information about plant species: local name, plant parts used, methods of preparation, administration and different diseases treated. Scientific names were given after botanical identification of harvested samples and listed in the table in alphabetical order. As indicated in the Table 1, 49 traditional healers allotted between twelve tribes, whose 16 women (32.65 %) and 33 men (67.34 %) were interviewed. Without accurate sociological information on these different tribes, it is not easy to explain clearly why there are more men traditional healers than women. However, we think that three reasons would explain that: (i) the will of advertising (use of poster, streamer, cartoon) that is more remarkable to men than to women; (ii) the fact that during the inquest time, more women than men are absent for field work would explain why there are more men traditional healers known than women; and (iii) it is possible that the practice of traditional medecine is guided by socio-cultural characteristics such as kinship system (patriarchy or matriarchy) as observed in the Mafa tribe of Cameroun [10]. As it can be observed the main source of traditional medicine knowledge remains the ancestral transmission way from old people to young ones (39/49). This may be explained by the fact that, traditional medicine is a cultural component which spread through generations from ascendants to descendants and based on oral transmission in Africa [8,11,12]. We notify that the Luba and Bemba tribes are the most representative tribes among the traditional practitioners respectively with 28.57% and 16.32%, only because they are the most numerous in the areas of inquiry [13] Annex 1. The information about the plants used in managing diabetes collected from different traditional medical practitioners is gathered in the following table. In this study, the data show that, 95 plants from 47 families were indicated as traditionally used to treat diabetes. Fabaceae (24.2%), Euphorbiaceae (7.4%), Apocynaceae and Loganiaceae (4.2 each) are the most representative botanical families. The ethnobotanical survey revealed that the root (41.3% of citation) is the most used organ of plant followed by the leaves (28.6%) and the stem bark (20.6%). The decoction found to be the main way to prepare recipes (62.2%) and the oral administration (92%) as the principal way to give antidiabetic traditional formulations. The present study showed that, apart from diabetes, the 95 plants mentioned by traditional healers are also used in the treatment of others several diseases or symptoms (more than forty) such as diarrhea, rheumatism, infections and abdominal pain. Each of the 95 plants cited was mentioned at least by one respond. Some species such as: *Albizia adianthifolia* (Schum.) WF Wight, *Antidesma venosu*m Meyer, *Cassia occidentalis* L, *Jatropha curcas* L and *Strychnos spinosa* Lam, were known as antidiabetic by two or more traditional healers (Annex 1).

**Table 1:** Information about the 49 traditional healers’ identity and source of knowledge

Site	N.A	Tribe and Sex	Age (year)	Source of knowledge
Kasumbalesa	6	Bemba 1F	49	Ancestral
		Kabinda 1F	52	Initiation of another tribe
		Luba 1M	70	Dreams
		Round 1F	49	Ancestral
		Tshokwe 1F; 1M	(71, 103)	Initiation of another tribe; Ancestral
Kipushi	16	Bemba 3M	(63, 63, 50)	Ancestral
		Kabinda 2M	(52, 70)	Ancestral
		Luba 2F; 2M	(68,48, 50, 36)	Ancestral; dreams
		Luba- Kasaï 3F; 2M	(59, 60, 49, 53, 69)	Ancestral; initiation of another tribe
		Rega 1M	51	Ancestral
		Tshokwe 1M	40	Ancestral
Likasi	5	Bemba 1F	41	Ancestral
		Luba 2M	71, 75	Ancestral; spirits
		Lunda 1F	75	Ancestral
		Tshokwe 1F	57	Spirits
Lubumbashi	22	Bemba 3M	53, 66, 50	Ancestral; spirits
		Bembe 2M	47, 40	Ancestral
		Hemba 1F; 3M	51, 88, 46, 50f	Ancestral
		Lélé 1M	27	Ancestral
		Luba 1F, 7M	42, 35, 45, 39, 55, 70, 55, 47	Ancestral; spirits; dreams
		Luba- Kasaï 1F; 1M	45, 60	Ancestral
		Sanga 1M	51	Ancestral
		Tshokwe 1F	41	Ancestral

N.A: Number of Answers (respond); M= male; F=female

**Annex 1:** Plants used traditionally in the management of diabetes at Kasumbalesa, Kipushi, Likasi and Lubumbashi

Scientific name	Local names	Family	U.P	Treated diseases	Preparation and administration	Site	**References**
*Acacia karroo* Hayne	Munga (Luba), Mutonge (Sanga)						
Mugunga (Hemba)	Fabaceae	Leave, Stem bark	Diabetes, vaginal infections, jaundice	Decoction/per os	Kasumbalesa	T41	
*Adansonia digitata* L.	Mululu punga (Bemba)	Bombaceae	Stem bark	Diabetes	Decoction/per os	Lubumbashi	T37
*Adenia gummifera* (Harv.) Harms	Komboponoke (Lamba), Kimboyi (Lala)	Passifloraceae	Stem bark	Diabetes, birth troubles, infections	Infusion/per os	Lubumbashi	T35
*Adenia venenata* Forssk.	Mafula (Luba)	Passifloraceae	Root, Leave,	Diabetes	Decoction/per os	Likasi	T4
*Afromosia angolensis* Harms.	Mubanga (Bemba), Mubanga kyulu (Luba)	Fabaceae	Root	Diabetes, abdominal pain	Decoction/per os	Kasumbalesa	T40
*Albizia adiantifolia* (Schum.) W. F. Wight	Kasikeaze (Tshokwe), Kapeta nzovu (Bemba), kapeta nzovu (Luba), Kampetanzevu(Tshiluba)	Fabaceae	Root	Diabetes, syphilis, diarrhoea, blennorroea, indigestion	Decoction/per os	Likasi, Kipushi, Lubumbashi	T7; T37; T42; T44;
*Allium cepa* L	Matungulu sumu (Swahili)	Alliaceae	Seed	Diabetes, High blood pressure	Maceration/per os	Kipushi	T36
*Allium sativum* L	Ail (Français)	Alliaceae	Bulb	Diabetes, abdominal pain	As a meal/per os	Lubumbashi	T2
*Aloe vera* L	Chigaka (Mashi)	Asphodelaceae	Leaves	Diabetes, dye, cancer	Maceration /per os	Lubumbashi	T19
*Ananas Comisus* Schult. F.	Nanasi (Swahili), Ananas (Français)	Bromeliaceae	Fruit	Diabetes, indigestion	Decoction/per os	Lubumbashi	T7
*Anisophyllea boehmii* Engl.	Fungo (Sanga), Lufunga (Tabwa)	Rhizophoraceae	Root	Diabetes, abdominal pain	Decoction/per os	Kipushi	T6
*Antidesma venosum* (Tul.) E. Mey.	Kifubia (Luba), Musambafwa (Lamba)	Euphorbiaceae	Stem bark	Diabetes, gastrite, blennorroea	Decoction/per os	Lubumbashi, Kasumbalesa	T18 ; T50
*Arachis hypogaea* Lam.	Mbaa (Bemba), mwema (Bembe)	Fabaceae	Leaves	Diabetes, infections	Decoction/per os	Lubumbashi	T5
A*ristolochia hockii* De Wild.	Kapanganganga (Bemba), Kazaz misang (Rund)	Aristolochiace ae	Root	Diabetes, measles, syphilis, dysmenorrhoea	Decoction/per os	Likasi	T8
*Asparagus africanus* Lam.	Mukoma wa kanyengelele (luba)	Aspagaraceae	Leaves Root	Diabetes, syphilis, haemorrhoid	Decoction/per os	Likasi, Kasumbalesa	T18 ; T33
*Azanza garckeana* (F. Hoffman) Excell & Hillc.	Muti ya makamashi (Swahili)	Malvaceae	Leaves Stem bark	Diabetes, œdema of the lower extremities, epilepsy	Decoction/per os Infusion/per os	Kasumbalesa	T40
*Balanites aegyptiaca* (L.) Delile	Mubamba ngoma (Swahili), Mbamba ngoma (Luba)	Balanitaceae	Root	Diabetes, sexual impotence, diarrhoea	Decoction/per os	Lubumbashi	T37
*Bidens pilosa* L.	Mpota ya mbwa (Luba), Asisa (Bembe)	Asteraceae	Leaves	Diabetes, hemostatic, urinary infections	Decoction/per os	Lubumbashi	T19
*Bougainvillea spectabilis* Willd	Bougainvillé (Français)	Nyctagynaceae	Flowers	Diabetes	Maceration/per os	Lubumbashi	T3
*Brassica oleracea* L.	Choux (Français)	Brassicaceae	Leaves	Diabetes, skin diseases	Infusion/per os	Kasumbalesa, Kipushi	T15 ; T36
*Brillantaisia patula* T. Anderson	Muleta (Zela)	Acanthaceae	Stem bark	Diabetes, gastrite	Decoction/per os	Lubumbashi	T45
*Canarium schweinfurthii* Engl.	Mpashi (Bemba), Mpafu (Luba)	Burseraceae	Leaves	Diabetes, haemorrhoid	Decoction/per os	Lubumbashi	T5
*Carica papaya* L.	Cimuti cha popo (Bemba), Kipawo (Sanga), Papai (Swahili)	Caricaceae	Leaves Root	Diabetes, worms, infections	Decoction/enema	Lubumbashi	T3 ; T9
*Cassia occidentalis (*L.) Link	Lukunda bajanyi (Tshiluba), Mbaw-mbaw (Kikongo)	Fabaceae	Seed Leaves Root	Diabetes, worms, constipation	Decoction,/per os Maceration/enema	Lubumbashi, Kipushi	T3 ;T27
*Cassia petersiana* Bolle.	Kafunga nashya (Bemba)	Fabaceae	Root	Diabetes, sexual impotence	Maceration/per os	Lubumbashi	T11
*Cassia sieberiana* DC.	Kandungandunga (Tshiluba), Mugunga (Hemba) Mununga nunsi (Bemba, Lamba).	Fabaceae	Leaves	Diabetes, worms	Decoction/per os	Lubumbashi	T31
*Catharanthus roseus* (L.) G.Don	Pervanche de Madagascar (Français)	Apocynaceae	Leaves Root	Diabetes, High blood pressure, worms, cough, malaria, cancer	Decoction/per os Maceration/enema	Kasumbalesa, Kipushi, Lubumbashi	T22 ; T23 ; T29
*Citrus limon* (L.) Burm. F.	Citronier (français)	Rutaceae	Root	Diabetes, cough, fever	Decoction/per os	Kasumbalesa	T15
*Citrus sinensis* Osbeck.	Ndimu (Swahili)	Rutaceae	Root	Diabetes, fever	Decoction/per os Maceration/enema	Lubumbashi	T11
*Coleus kilimandschari* Guerke.	Mcubya (Bembe), Mutozo (Shi), Mulavumba (Swahili)	Lamiaceae	Leaves Root	Diabetes, haemorrhoid, malaria, abdominal pain, malaria, cough, angine, diabetes	Decoction/per os Infusion/per os Maceration/per os	Kipushi	T23 ; T27
*Combretum celastroides* Sensu Exell &Garcia	Lukondambo (Luba), Mwina kyulu (Sanga)	Combretaceae	Leaves Stem bark	Diabetes, skin diseases	Decoction/per os	Kipushi	T16
*Crossopteryx febrifuga* (G.Don)Benth.	Mutoshi (Tshiluba), Konsekonse (Lamba, Bemba)	Rubiaceae	Leaves Root	Diabetes, abdominal pain	Maceration/per os	Lubumbashi	T39
*Crotalaria spinosa* (Benth) Hutch.	Kabalala (Sanga)	Fabaceae	Stem bark Root	Diabetes, venereal diseases	Decoction/per os	Kipushi	T21
*Croton macrostachyus* (Delile) Hochst.	Mutara mutshi (Bemba)	Euphorbiaceae	Leaves	Diabetes, blennorroea, dysmenorrhoea	Decoction/enema	Likasi	T4
*Cucumis sativus* L.	Concombre (Français)	Cucurbitaceae	Fruit	Diabetes	As a meal/per os	Lubumbashi	T12
*Cyperus alternifolius* L.	Ndao (Luba)	Cyperaceae	Stem bark	Diabetes, asthma, abdominal pain	Decoction/per os	Lubumbashi	T35
*Dalbergia boehmii* Taub.	Katembo mutshi (Luba-kassai), Katembo (Zela, sanga)	Fabaceae	Leaves Stem bark	Diabetes, abdominal pain, rheumatism, diarrhoea, carie dentaire, abortion threat	Decoction/per os	Lubumbashi Kipushi	T 30 ; T48
*Diplorhynchus condylocarpon* (Muell.Arg) Pichon.	Mwenge (Swahili)	Apocynaceae	Root	Diabetes, blennorroea	Decoction/per os	Lubumbashi	T37
*Droogmansia munamensis* De Wild.	Mununganunga (Bemba), Mulundeni (Lala)	Fabaceae	Leaves Stem bark	Diabetes, dysentery	Decoction/per os	Lubumbashi	T38
*Elaeis guineensis* Jacq.	Ekaci (Bembe)	Arecaceae	Root	Diabetes, sterility	Decoction/per os	Lubumbashi	T31
*Entada abyssinica* Steud.ex A.Rich) Gilbert	Kipungu (Sanga)	Fabaceae	Root	Diabetes, haemorrhoid	Decoction/per os	Lubumbashi	T7
*Erythrina abyssinica* Lam.	Kisongwa (Hemba) ; Kisungwa (Bemba	Fabaceae	Root	Diabetes	Decoction/per os	Kasumbalesa Lubumbashi	T22 ; T45
*Erythrophleum africanum* (Benth.) Harms	Kayimbi (Tshiluba)	Fabaceae	Leaves Stem bark	diabetes, cancer, rheumatism	Decoction/per os Maceration/per os	Kipushi	T20
*Faurea saligna* Harv.	Mulemu (Sanga)	Proteaceae	Root	diabetes	Decoction/per os	Kipushi	T21
*Ficus sycomorus* L.	Mukunyu (Swahili), Tshikuyi (Luba)	Moraceae	Leaves Stem bark Root	Diabetes, Diarrhoea	Decoction/per os	Kipushi Lubumbashi	T25 ; T39
*Garcinia huillensis* (Oliv.)Welw.	Mungindu (Tchokwe)	Clusiaceae	Root	Diabetes, rheumatism, gastro-intestinal troubles	Decoction/per os	Kasumbalesa	T34
*Gladiolus klattianus* Hook.	Kitala (Bemba), Kitokatoka (Luba)	Iridaceae	Bulb	Diabetes, blennorroea, fever	Maceration/per os	Lubumbashi	T38
*Glycine max* (L.) Merr.	Soja (swahili)	Fabaceae	Leaves	Diabetes	Decoction/per os	Lubumbashi	T31
*Grewia flava* DC.	Bungwe (Luba)	Tiliaceae	Leaves Stem bark	Diabetes, hernia	Decoction/per os	Kasumbalesa	T18
*Harungana madagascariensis* Lam.ex Poir.	Mukuta (Tshiluba)	Hypericaceae	Stem bark Root	Diabetes, rheumatism, High blood pressure	Decoction/per os, enema	Kipushi	T13
*Hymenocardia acida* Tul.	Kapembe (Bemba), Ambalanga (Hemba), Lupep (Tshokwe)	Hymenocardiaceae	Root	Diabetes, haemorrhoid	Decoction/per os	Lubumbashi, Kasumbalesa	T37 ; T16
*Ipomoea spathulata* Hallier.f.	Mulapa (Sanga)	Convolvulaceae	Leaves	Diabetes, worms	Chewing/per os	Lubumbashi	
*Jatropha curcas* L.	Mbono (Swahili), Ntondondimba (Bemba), Kilembelembe (Luba)	Euphorbiaceae	Leaves Seed Root	Diabetes, gastrite, fricanae, urinary infections	Pression/per os	Lubumbashi , Kipushi,	T32; T50; T46; T17; T2
*Justicia flava* (Forssk.) Vahl	Luhe (Luba)	Acanthaceae	Stem bark	Diabetes, dysmenorrhoea, amibiase	Decoction/per os	Lubumbashi	T48
*Kigelia africana* (Lam) Benth.	Kivungu (Luba)	Bignoniaceae	Stem bark	Diabetes, sexual impotence, Vaginal diseases	Decoction/per os	Lubumbashi	T48
*Lantana camara* L.	Mavi ya kuku (Swahili)	Verbenaceae	Leaves	Diabetes, fever, cough, cephalgia	Decoction/per os Infusion/per os	Likasi	T8
*Lonchocarpus katangensis* De Wild.	Chuya (Bemba)	Fabaceae	Stem bark	Diabetes, syphilis, dental carie	Maceration/per os	Kasumbalesa	T41
*Maesopsis eminii* Engl.	Ndunga (Luba)		Leaves Stem bark	Diabetes, eye troubles, urinary infections	Decoction/per os	Kipushi	T27
*Maprounea africana* Müll .Arg.	Kafula ndime (Luba)	Euphorbiaceae	Root	Diabetes, vaginal pain	Decoction/per os	Kipushi	T17
*Maytenus senegalensis* (Lam.) Exell	Tshingala mutshi (Luba)	Celastraceae	Leaves Root	Diabetes, diarrhoea	Decoction/enema	Lubumbashi	T9
*Mucuna poggei* Taub.	Mpesa (Tshiluba)	Fabaceae	Root	Diabetes	Decoction/per os	Lubumbashi	T51
*Musa sapientum* L.	Bananier (français)	Musaceae	Bulb	Diabetes, rheumatism	Decoction/per os	Lubumbashi	T31
*Olax obtusifolia* De Wild .	Kulokumo (Bemba)	Olacaceae	Root	Diabetes, paralysis	Decoction/per os	Lubumbashi	T37
*Opuntia ficus-indica* (L.)Mill.	Cactus (Français)	Cactaceae	Leaves	Diabetes, haemorrhage	Chewing/per os	Lubumbashi	T50
*Persea americana* Mill.	Ikipapai (Lamba), Avocatier (Français)	Lauraceae	Leaves Stem bark	Diabetes, fever, anemia	Decoction/per os	Kasumbalesa, Kipushi	T17 ; T40
*Phaseolus lunatus* L.	Haricot (Français), Maharagi (swahili)	Fabaceae	Leaves Seed	Diabetes, abdominal pain	Décoction/per os Infusion/per os	Kipushi, Lubumbashi	T11 ; T14
*Piliostigma thonningii* (Schumach.)Milne-Redh.	Kifumbe (Bemba, Luba)	Fabaceae	Root	Diabetes, cough, anemia	Maceration/per os	Kasumbalesa	T22 ; T45
*Protea obtusifolia* Oliv.	Mwinkala nikata (Tabwa)	Proteaceae	Root Stem bark	Diabetes	Decoction/per os, enema	Lubumbashi	T43
*Pseudolachnostylis maprouneifolia* Pax.	Musangati (Swahili), Musangali (Bemba), Musaria (Tchokwe)	Euphorbiaceae	Leaves Root	Diabetes, gastrite, digestion troubles, cough, diarrhoea, dysméenorrhoea	Decoction/per os Chewing/per os	Lubumbashi, Kasumbalesa	T34 T37
*Psidium guajava* L.	Lipela (Swahili)	Myrtaceae	Leaves Root	Diabetes, dysenterie	Decoction/per os Maceration/per os	Lubumbashi	T51
*Pterocarpus angolensis* DC.	Mukundambazu (Tabwa), Muyanga (Bemba)	Fabaceae	Stem bark	Diabetes, haemorrhoid	Decoction /per os	Likasi	T4
*Pterocarpus tinctorius* Welw.	Mukula (Chokwe)	Fabaceae	Root	Diabetes	Decoction/per os	Kasumbalesa	T34
*Rauwolfia caffra* Sond.	Mutalala (Bemba)	Apocynaceae	Leaves Stem bark	Diabetes, malaria, snake’s bites	Decoction/per os	Kasumbalesa	T23
*Rauwolfia vomitoria* Afzel.	Pandanganga (Luba)	Apocynaceae	Root	Diabetes, purgative	Decoction/per os	Kipushi	T17
*Rhynchosia insignis* (O.Hoffm.) R.E.Fr. .	Munkoyo (swahili)	Fabaceae	Root	Diabetes, jaundice	Maceration/per os	Kasumbalesa	T22
*Ricinus communis* L.	Lundimba ndimba (Luba), Mubalika (Bemba)	Euphorbiaceae	Root	Diabetes	Decoction/per os	Lubumbashi	T46
*Sesamum angolense* Welw.	Kipalabwengo (Bemba)	Pedaliaceae	Root	Diabetes	Decoction/per os	Lubumbashi	T37
*Solanum seretii* De Wild.	Impwa (Bemba)	Solanaceae	Root	Diabetes, abdominal pain	Decoction/per os enema	Lubumbashi	T47
*Solanum subsessile* De Wild.	Mutete (Luba)	Solanaceae	Leaves Seeds	Diabetes, abdominal pain	As meal/per os	Lubumbashi	T32
*Solanum tuberosum* L.	Pomme de terre (français)	solanaceae	Tubercule	Diabetes, anti acid	As a meal /per os	Lubumbashi	T2
*Strychnos cocculoides* Baker.	Katongatonga (Luba), Bukoke (Hemba), Kisongole (Bemba)	Loganiaceae	Root	Diabetes, abdominal pain, dysentery	Decoction/per os Infusion/per os	Kasumbalesa, Lubumbashi	T41; T32; T35
*Strychnos innocua* Delile.	Kakomekone (Swahili)	Loganiaceae	Root	Diabetes, blennorroea	Decoction/per os	Lubumbashi	T32
*Strychnos spinosa* Lam.	Kisongole (Bemba), Nsansa (Swahili)	Loganiaceae	Stem bark Root	Diabetes, blennorroea	Decoction/per os	Lubumbashi, Kipushi,	T1; T24; T37; T44; T50
*Strychnos stuhlmannii* Gilg.	Mubanga Kyilu (Bemba), Nkanga kyulu (Zela)	Loganiaceae	Root	Diabetes, Gangrene, syphilis	Decoction/per os	Lubumbashi	T12 ; T24
*Swartzia madagascariensis* Desv.	Munienze (Luba), Mpampi (Tshiluba)	Fabaceae	Root	Diabetes, Touthache	Decoction/per os	Kipushi, Lubumbashi	T26 ; T50
*Syzygium guineense* (Willd) DC.	Musanfwa (Bemba)	Myrtaceae	Stem bark	Diabetes	Decoction/per os	Likasi	T42; T43
*Terminalia mollis* M.A. Lawson.	Kianga (Hemba), Tshibangu Mutshi (Tshiluba)	Combretaceae	Leaves Root	Diabetes, diarrhoea, syphilis	Decoction/per os	Lubumbashi, Kasumbalesa	T40
T28							
*Tithonia diversifolia* (Hemsley.) A.Gray.	Bilombalomba (Lélé)	Asteraceae	Leaves	Diabetes, abdominal pain	Chewing/per os Maceration/per os	Lubumbashi	T32
*Uapaka kirkiana* Müll. Arg.	Masuku (Bemba, Luba)	Euphorbiaceae	Stem bark	Diabetes, diarrhoea, sterility, Headache	Decoction/per os	LIkasi	T8
*Vernonia shirensis* Oliv. & Hiern.	Kilulukunja (Swahili), Muvurumen (Rund)	Asteraceae	Leaves Root	Diabetes, haemorrhoid, worms	Decoction/per os	Kasumbalesa Lubumbashi	T23 T2 ; T1
*Vigna sinensis* A.Rich	Lukunde (kikabinda)	Fabaceae	Leaves Root	Diabetes, headache	Decoction/per os Maceration/per os	Kipushi	T36
*Vitex madiensis* Oliv.	Mufutu (Luba)	Verbenaceae	Leaves Root	Diabetes	Decoction/per os	Kipushi	T34 ; T37
*Vitis vinifera* L.	Raisin (Français)	Ampelidaceae	Leaves	Diabetes	Decoction/per os	Lubumbashi	T50
*Zanthoxylum chalybeum* Engl.	Mpupwe kiulu (Luba), Pupwe (Bemba)	Rutaceae	Leaves Stem Root	Diabetes, gastrite, cough, otitis, hip pain, sterility	Decoction/per os	Lubumbashi Kipushi, Likasi	T10 ; T24 ; T49
*Ziziphus mucronata* Willd.	Kankona (Luba, Bemba, sanga)	Rhamnaceae	Stem bark Root	Diabetes, dysentery, abdominal pain	Decoction/per os	Likasi	T33

## Discussion

This is a first report of an ethnobotanical survey of species used as antidiabetic in the study area. The predominance of Fabaceae, Euphorbiaceae as major botanical families comprising more species used in traditional medicine was also mentioned in a similar study in the same area [14,15]. This study has shown that the root is the most widely used organ for the preparation of recipes. Cheikhyoussef et al [16] as well as Tabuti et al [17], found also in their studies that root and leaves have been more used than other plant organ. The large use of decoction and oral administration respectively as the main preparation mode and the principal route to give traditional herbal drugs are a generally observed in other African communities. The use of the different plants in the management of diabetes and other ailments demonstrates the importance of traditional medicine that is known to be a component of everyday life in many areas of the world and particularly in Africa [8,18]. When comparing this study with others, some resemblance can be pointed out: among 306 vegetables species cited as antidiabetic plants used in the treatment of diabetes in Mexico [19], 11 plants are identified in our study: *Allium cepa* L, *Aloe vera* L, *Ananas comosus* L, *Arachis hypogeal* L, *Bidens pilosa* L, *Carica papaya* L, *Catharanthus roseus* L, *Persea Americana* Mill, *Psidium guajava* L, *Ricinus cominus* L, *Senna occidentalis* L as used by traditional healers in the magament of diabetes. In the ethnobotanical investigation conducted by Abo, Fred-Jaiyesimi and Jaiyesimin in the South Western Nigeria area [20], 31 plants had been reported to be used traditionally as antidiabetic agents and *Carica papaya* cited in our study is revealed in that study. *Allium cepa*, *Allium sativum*, *Bidens pilosa*, *Catharanthus roseus*, *Lantana camara*, *Musa sapientum* and *Psidium guajava*identified in this investigation are documented as antidiabetic used traditionnaly in other studies [21,22]. The antidiabetic properties of some species identified in this investigation have been experimentally demonstrated in the in vivo and in vitro diabetic models: *Allium cepa*, *Allium sativum* [23], *Aloe vera*, *Bidens pilosa* [19,24]; *Catharanthus roseus* [25-27], *Lantana camara* [23,28], *Musa sapientum* [29,30]. Compared to another ethnobotanical survey of plants used as antidiabetic in Kisangani, Eastern province of DRC, 10 species cited in this study are also mentioned by Katemo et al [31].

## Conclusion

In this study tradipraticians cited both medicinal herbs already known for their antidiabetic effect (34 plants, 35.8% of citations) and so far uncited herbs that must be evaluated for hypoglycemic and antihyperglycemic and other diabetic related symptoms; so that they may possibly be used in the management of diabetes.

### What is known about this topic

- For this topic, it is known that the population of Lubumbashi and its surroundings uses traditional medicine to treat various diseases. It is also known that in most cases, this traditional medicine exploits plant resources as a source of medicines. Some of these plants are used in the treatment of diabetes.

### What this study adds

- The novelty of this study is summarized in that: (i) this study lists for the first time the plants used against diabetes in Lubumbashi and its surroundings; (ii) Among the inventoried species, some have not yet been studied in this field and are probably a particularity of Congolese traditional medicine; (iii) For the first time, the profile of providers of traditional diabetes care in Lubumbashi is given.

## Competing interests

The authors declare no competing interests.
